# The Preparation and Microstructure of Nanocrystal 3C-SiC/ZrO_2_ Bilayer Films

**DOI:** 10.3390/nano7120408

**Published:** 2017-11-23

**Authors:** Chao Ye, Guang Ran, Wei Zhou, Yazhou Qu, Xin Yan, Qijin Cheng, Ning Li

**Affiliations:** 1 College of Energy, Xiamen University, Xiamen 361102, China; kim.yc@foxmail.com (C.Y.); qyz1991@live.com (Y.Q.); xinyan3056@outlook.com (X.Y.); qijin.cheng@xmu.edu.cn (Q.C.); 2 China Academy of Engineering Physics, Mianyang 621900, China; zhouwei_801202@163.com

**Keywords:** 3C-SiC/ZrO_2_ film, thin film, nanomaterials, PECVD

## Abstract

The nanocrystal 3C-SiC/ZrO_2_ bilayer films that could be used as the protective coatings of zirconium alloy fuel cladding were prepared on a single-crystal Si substrate. The corresponding nanocrystal 3C-SiC film and nanocrystal ZrO_2_ film were also dividedly synthesized. The microstructure of nanocrystal films was analyzed by grazing incidence X-ray diffraction (GIXRD) and cross-sectional transmission electron microscopy (TEM). The 3C-SiC film with less than 30 nm crystal size was synthesized by Plasma Enhanced Chemical Vapor Deposition (PECVD) and annealing. The corresponding formation mechanism of some impurities in SiC film was analyzed and discussed. An amorphous Zr layer about 600 nm in width was first deposited by magnetron sputtering and then oxidized to form a nanocrystal ZrO_2_ layer during the annealing process. The interface characteristics of 3C-SiC/ZrO_2_ bilayer films prepared by two different processes were obviously different. SiZr and SiO_2_ compounds were formed at the interface of 3C-SiC/ZrO_2_ bilayer films. A corrosion test of 3C-SiC/ZrO_2_ bilayer films was conducted to qualitatively analyze the surface corrosion resistance and the binding force of the interface.

## 1. Introduction

Zirconium alloys are mainly used as nuclear fuel claddings and fuel assemblies in pressurized water reactors operating at 300 ± 50 °C [1,2]. However, in service, aqueous corrosion and hydrogenation of zirconium alloys are a serious problem and represent a nuclear safety hazard [3,4,5]. Along with the extended reload cycle of the fuel assembly, increased fuel burnup, and zero tolerance on nuclear accidents, the development of new types of fuel claddings has become an area of great interest in recent years. There are two major popular solutions, including: (1) to develop new kinds of nuclear fuel claddings such as SiC/SiC_f_ composites [6], FeCrAl alloys [7] and Mo alloys [8], and (2) to develop zirconium alloy fuel cladding with a protective coating on its outside surface. The function of surface coating is used to isolate zirconium-water reactions under conditions of high temperature and strong irradiation. Because of its high-temperature stability, excellent irradiation resistance, low neutron capture cross section and non-reaction with water at high temperature, SiC will be an ideal candidate for use as the coating of zirconium alloys. Compared with SiC with other crystal structures, β-SiC (also named 3C-SiC) has better anti-wear, lower processing temperature, and higher corrosion resistance and irradiation resistance [9]. Meanwhile, 3C-SiC has been successfully used as the middle layer for preventing fission product diffusion and providing structural support in TRISO (Tri-Structural Isotropic) fuel particles [10].

There are several kinds of methods to prepare SiC thin film, including heating evaporation [11], arc discharge [12], carbon reduction [13], sol-gel [14], chemical synthesis [15] and floating catalyst [16]. The widely used methods are chemical vapor deposition (CVD) [17,18,19,20] and plasma-enhanced CVD (PECVD) [21,22,23]. The Plasma Enhanced Chemical Vapor Deposition (PECVD) process does not need a high deposition temperature and can greatly accelerate the deposition rate. Semenov [24] prepared 3C-SiC film through the direct deposition of carbon and silicon ions. The content of the nanocrystalline 3C-SiC phase was up to 80% in the film at 950 °C. However, when the temperature was over 1000 °C, the crystal structure would be changed from cubic to rhombohedral to form a α-SiC-21R phase [24]. Oliveira [25,26] also synthesized 3C-SiC film using the PECVD technique. In fact, it has been found that even tiny amounts of impurities are likely to affect the performance of 3C-SiC film. However, there are few articles relating with the formation mechanism of impurities in the 3C-SiC film.

Because of the large difference of lattice constants between 3C-SiC and the α-Zr matrix, there will be a huge lattice mismatch, which will induce large internal stress and make a weak binding force on the interface. Therefore, a transition layer between the SiC film and Zr matrix become very important to decrease the lattice mismatch degree and to improve the interfacial properties. Therefore, ZrO_2_ will be selected as a candidate of transition layer between 3C-SiC and Zr matrix.

In the present work, the investigation of the nanocrystal 3C-SiC thin film and nanocrystal ZrO_2_ thin film grown severally on single crystal Si substrates was carried out to obtain an optimal preparation process parameter. Then, the preparation of 3C-SiC/ZrO_2_ bilayer films and their interface characteristics were researched. The microstructure of the prepared nanocrystal thin films was analyzed and the correspondingly mechanism was also discussed.

## 2. Experiments

### 2.1. Sample Fabrication

**Figure 1:**
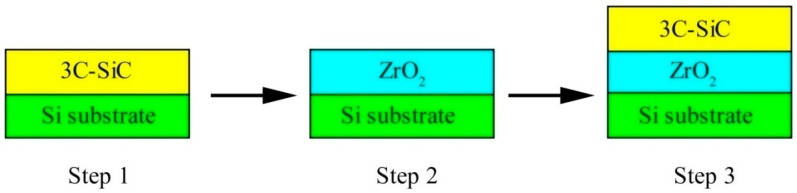
The flow chart of preparation process of 3C-SiC/ZrO_2_ thin film.

The flow chart of preparation process of nanocrystal 3C-SiC/ZrO_2_ thin film in the present work is shown in Figure 1. Firstly, the nanocrystal 3C-SiC thin film grown on a single crystal Si substrate with different preparation parameters was investigated to obtain an optimal preparation process parameter. Secondly, the nanocrystal ZrO_2_ thin film that can be used as transition layer between zirconium alloy fuel cladding and SiC protection layer was deposited on a single crystal Si substrate in order to obtain an ideal preparation parameter. The application aim of the nanocrystal ZrO_2_ transition layer is to decrease the lattice mismatch degree between 3C-SiC thin film and Zr matrix. Finally, the 3C-SiC/ZrO_2_ bilayer films grown on a single crystal Si substrate were prepared under two different annealing processes. The detail preparation process and corresponding experiment parameters were described as follows.

(1) Step one: The precursor gases of methane (CH_4_) and silicane (SiH_4_) were used to deposit a-SiC: H (amorphous SiC after hydrogenation) thin films by using Plasma Enhanced Chemical Vapor Deposition (PECVD) System (PECVD 350 series, Beijing Tai Ke Nuo Company, Beijing, China) with a radio frequency (RF) generator of 13.56 MHz and PLC (Programmable Logic Control) control mode. The hydrogen (H_2_) was used to smooth the surface of deposited films. Firstly, only CH_4_ gas was used to offer carbon ions to react with single crystal Si substrate with [100] direction in order to form a-SiC: transition layer. Then, a-SiC: H thin films under the conditions of a different ratio of CH_4_ to SiH_4_ and different RF powers were deposited on the a-SiC: transition layer at 400 °C for 30 min. The pressure in the deposition chamber was set to 0.2 Torr. After being deposited, the samples were annealed for 1.5 h at 1000 °C in a tube furnace with the protection of 20 sccm (standard-state cubic centimeter per minute) argon (99.999% purity) flow rate. A method was used to decrease oxygen content during the annealing process, which was that before annealing the samples were previously loaded into a corundum can, which was sealed using Al_2_O_3_ adhesion agent in the glove box protected by high pure argon gas. The oxygen content was less than 12 ppm in the glove box, so the samples were only exposed at an environment with very low oxygen content. The experimental parameters of deposition and annealing used to prepare the nanocrystal 3C-SiC thin film were listed in Table 1.
(2) Step two: A Zr layer with a thickness of about 500 nm was first deposited on a single crystal Si substrate with [100] direction using magnetron sputtering system, and then annealed for 1.5 h at 1000 °C in a tube furnace with a low-speed flow argon protection. The target material used in magnetron sputtering is a circular α-Zr target produced by JAH Technology Company (Beijing, China) with the diameter of 50.8 mm, a purity of 99.9%, and a thickness of 5 mm. The magnetron sputtering system is a Desktop Pro series product of the Denton company (Denton, TX, USA), which is equipped with RF/DC (Direct Current) sputtering power and can drive two target guns. It is based on a PLC control system and can reach 5% of the coating uniformity. Because of the low gas tightness of tube furnace and low-speed flow argon, at high temperature the deposited Zr layer could be easily oxidized to form zirconium oxides at rare oxygen conditions, especially for the amorphous zirconium. Therefore, it did not need to be exposed to the atmosphere to form zirconium oxide film at high temperature annealing. The experimental parameters of deposition and annealing used to prepare the nano-polycrystalline ZrO_2_ thin film were listed in Table 2.
(3) Step three: For the preparation of the 3C-SiC/ZrO_2_ bilayer films, two different annealing schemes were used in the present work including: (i) Firstly, ZrO_2_ thin film was prepared on the single crystal Si substrate as mentioned in step two. Then, 3C-SiC thin film was prepared on ZrO_2_ layer as mentioned in step one. Thus, the whole preparation process has undergone two separate annealing processes. In order to conveniently express this in the next content, the T-3C-SiC/ZrO_2_ was used to represent the prepared thin film; (ii) the Zr layer was deposited on a single crystal Si substrate firstly, and then SiC layer was deposited on the as-deposited Zr layer surface. Finally, the deposited amorphous SiC/Zr bilayer films were annealed for 1.5 h at 1000 °C in a tube furnace with a low-speed flow argon protection. The O-3C-SiC/ZrO_2_ was used to represent the abovementioned thin film. After being annealed, amorphous SiC/Zr bilayer films were changed to nanocrystal 3C-SiC/ZrO_2_ bilayer films. The deposited amorphous Zr layer was oxidized to ZrO_2_ layer even at the coverage of SiC layer. This preparation process only underwent one annealing process. The microstructure and chemical composition of the interface between 3C-SiC and ZrO_2_ layers prepared by the above two methods were characterized and analyzed.

### 2.2. Microstructural Analysis

The microstructural phase of the deposited films was investigated by a Rigaku D/max-3C X-ray diffractometer with CuK_α_ radiation (λ = 0.1540598 nm). This X-ray diffractometer was produced by Rigaku Company (Tokyo, Japan) with a tube voltage of 40 kV and tube current of 30 mA. Grazing incidence X-ray diffraction (GIXRD) mode was used for scanning. The incident angle of X-ray was set as a fixed angle α, while the receiving angle was set to a range from 20° to 50°. The detected depth at a fixed angle of GIXRD could be calculated using the total reflection model [27] that gave a quantitative relationship between X-ray penetration depth and the incident angle (α), which would be ensured that the detection position was located in the deposited film.

Cross-sectional transmission electron microscopy (TEM) samples prepared by a method of mechanical thinning and then ion milling were used to analyze the microstructure of deposited films using a JEOL 2100 transmission electron microscope produced by JEOL Company (Tokyo, Japan) with 200 kV working voltage. The TEM sample was first cut from Si bulks with deposited films, along with the deposition direction, and then mechanically polished to about 15 μm thickness with diamond sandpapers. Sample was glued on a copper grid by G-1 epoxy glue and finally thinned to about 100 nm thickness via Ar ion milling using a Gatan 695 PIPS produced by the Gatan Company in Hong Kong, China. The double ion beam mode was used to thin the samples. Firstly, the energy of 6.5 V and 6° angle were used for quick thinning, and then the energy of the 3.5 V and incidence angle of 2° for fine thinning.

## 3. Results and Discussion

### 3.1. Microstructure Analysis of 3C-SiC Thin Film

**Figure 2:**
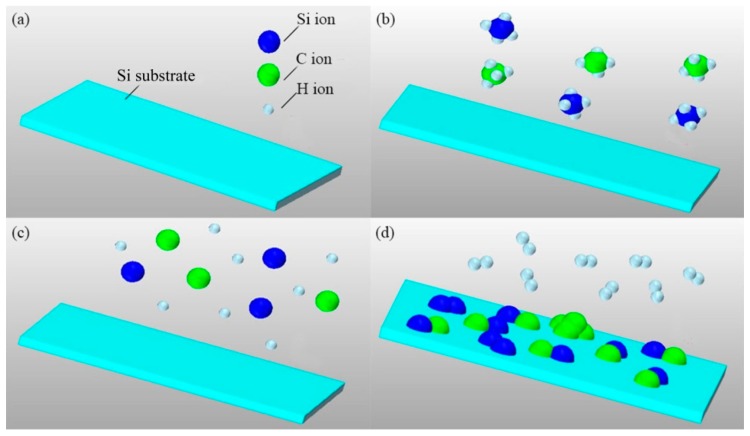
The sketch of preparation process of nanocrystal 3C-SiC thin film using Plasma Enhanced Chemical Vapor Deposition (PECVD). (**a**) The Si substrate; (**b**) SiH_4_ and CH_4_ are inputted into the reaction vessel; (**c**) The reaction gases are decomposed into the ions such as H^+^, Si^+^ and C^+^; (**d**) The ions combine to form neutral species, such as SiC, H_2_,Si and C, which are adsorbed on the surface of the Si substrate. The H_2_ gas is pumped out of the vessel with other unreacted gas.

The preparation process of nanocrystal 3C-SiC thin film mainly includes deposition and annealing processes. During the PECVD deposition process, SiH_4_ and CH_4_ are first inputted into the reaction vessel with H_2_. Under the action of glow discharge plasma, the reaction gases can be decomposed into ions such as H^+^, Si^+^ and C^+^. The temperature of electron gas is much higher than that of the ordinary gas molecule. These ions can combine to form neutral species, such as SiC, Si and C, that are easily adsorbed on the surface of the silicon substrate with low temperature. The chemical reactions among these neutral species will form a nanocrystal thin film. The sketch of above deposition process is shown in Figure 2. The chemical reaction equation can be written as follows:

SiH_4_(g) + CH_4_(g) → SiC(s) + 4H_2_(g) (1)

As mentioned above, the reaction gases in the ionization process are first decomposed to Si^+^, C^+^ and H^+^ ions. The species such as Si, C, Si*_x_*C*_y_* (*x* and *y* indicating chemical content) will be formed according to the random combination of Si^+^ with Si^+^, C^+^ with C^+^, and C^+^ with Si^+^. As a result, during the deposition process, it is inevitable to form impurity species such as Si, C and Si*_x_*C*_y_*. Therefore, an attempt should be made to have Si^+^ and C^+^ combine more easily following the proportion of 1:1 in the plasma environment in order to reduce the combination probability of same ions and different ratio of C^+^ to Si^+^ (not 1:1). Table 3 shows the bond energy of the substance involved in the reaction [28]. For the reason that if the bond energy of compound is higher, the compound will be more stable. Once it is formed, it is not easy to be broken down. So, according to Table 3, most of H^+^ will recombine with Si^+^, C^+^ or themselves to generate C*_x_*H*_y_*, Si*_x_*H*_y_* and H_2_ gases that can be discharged from the reaction vessel with the main airflow by vacuum pump. Most of the rest Si^+^ ions will combine with C^+^ ions to form SiC. Because the bond energy of C–H is high, CH_4_ is difficult to ionize, which will result in the concentration of C^+^ being relatively low in the plasma environment when the concentration difference between CH_4_ and SiH_4_ is not very big. Faced with the relatively larger concentration of Si^+^, C^+^ is more likely to meet with Si^+^ rather than C^+^. Therefore, the ratio of SiH_4_ to CH_4_ will play an important role influencing on the formation of the impurities in the nanocrystal thin film.

According to thermodynamic equilibrium conditions, plasma environments can be divided into three categories [29]: (1) complete thermal equilibrium (CTE); (2) local thermodynamic equilibrium (LTE), and; (3) non-local thermodynamic equilibrium (NLTE). In the LTE category, the plasma collision process is dominant. The collision process and inverse process satisfy the detailed balance condition. The physical exercise time is not less than that of the chemical equilibrium time. Therefore, the ions in motion have enough time to achieve a balance. Based on this viewpoint, the plasma environment of LTE can be described by the “double temperature model”, which is a simulation model with *T_e_* (temperature of electron) and *T_h_* (temperature of heavy particle) variables. According to the characteristics of the plasma environment of the PECVD reaction vessel in the present work, it should belong to the LTE category.

**Figure 3:**
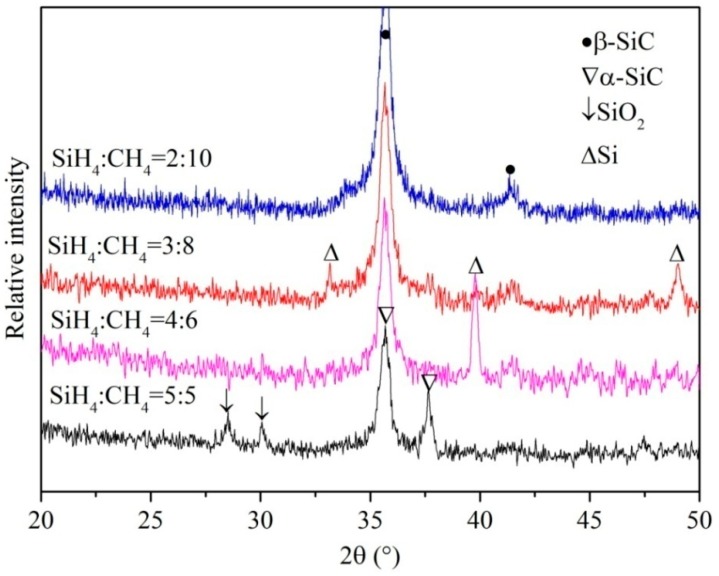
X-ray diffraction (XRD) patterns of the annealed thin films deposited with different ratios of SiH_4_ to CH_4_.

Figure 3 shows X-ray diffraction (XRD) patterns of the annealed SiC films deposited with different ratio of SiH_4_ to CH_4_. The nanocrystal thin film with the entire β-SiC phase is the ultimate goal in the present work. From the XRD results, it can be seen that the ratio of SiH_4_ to CH_4_ obviously influence the final phases of the annealed thin films. The XRD results are obvious different, although all experimental parameters, except for the ratio of original gases, are the same. In the XRD pattern of the #4 sample (SiH_4_:CH_4_ = 5:5) in Table 1, the peaks of α-SiC (JCPDS#39-1196 Rhombohedral SiC 2θ = 35.695°, 37.670°) with a rhombus crystal structure can be observed. The diffraction peaks of SiO_2_ (JCPDS#70-3315 Hexagon SiO_2_ 2θ = 28.285°) phase with very low intensity can also be detected. Importantly, no diffraction peaks of β-SiC can be found. Therefore, at the condition of SiH_4_:CH_4_ = 5:5, it is difficult to synthesize 3C-SiC thin film. A.V. Semenov [24] indicated that the polytypes of epitaxial growth SiC were a function of the concentration of the carbon vacancies *V_c_* in the grown layer. When the concentration of carbon vacancies is decreased, the degree of polytype hexagonality (γ) is sequentially increased in the following way: 6H-SiC (γ = 0.33); 15R-SiC (γ = 0.40); 4H-SiC (γ = 0.50), and; 2H-SiC (γ = 1); this means that the cubic structure with the minimum hexagonality γ = 0 turns out to be the most advantageous form from the viewpoint of energy when the *V_c_* is surplus. During the deposition process, carbon deficiency in the cubic SiC polytype will be formed in the grown layer. Taking into account the relevant models proposed by other researchers [24,30,31,32,33,34,35,36,37,38,39], when CH_4_ flow rate is decreased, the content of carbon vacancies will also be reduced, which will result in the degree increase of polytype hexagonality to make the sample itself more stable.

When the ratio of SiH_4_ to CH_4_ are located at the range from 2/10 to 4/6, the diffraction peaks of 3C-SiC (JCPDS#29-1129 Cubic SiC 2θ = 35.597°) can be observed in the XRD spectrums, as shown in Figure 3. However, the Si peaks (JCPDS#35-1158 Cubic Si 2θ = 39.9°) can also be seen when the ratio of SiH_4_ to CH_4_ is at the range from 3/8 to 4/6, it may be due to the effect of the Si substrate or the generated Si impurities in the thin film. Therefore, the ratio of SiH_4_:CH_4_ = 2:10 is an optimal compositional ratio and used in the following experiment.

**Figure 4:**
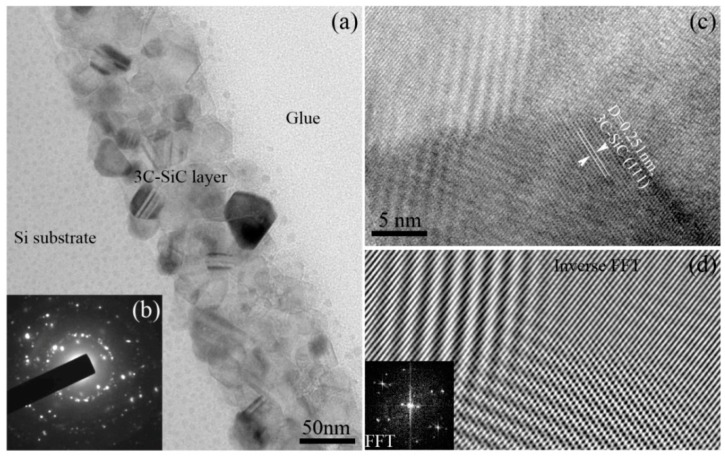
Bright field transmission electron microscopy (TEM) images and selected area electron diffraction (SAED) pattern of the annealed 3C-SiC thin film prepared from the mixture gases of SiH_4_:CH_4_ = 2:10, (**a**) The microstructure of 3C-SiC film; (**b**) SAED pattern of deposited film; (**c**) High-resolution TEM (HRTEM) image showing the grain boundaries; (**d**) Inverse FFT image of the HRTEM image of (**c**).

Figure 4 are the TEM images and selected area electron diffraction (SAED) pattern of the annealed 3C-SiC film prepared from the mixture gases of SiH_4_:CH_4_ = 2:10. It can be seen that a polycrystalline 3C-SiC layer with about 100 nm thickness is formed on the single crystal Si substrate. The average grain size is less than 30 nm. In addition, the thickness of 3C-SiC thin film is fairly homogeneous. The SAED pattern of the deposited film is shown in Figure 4b, which shows the typical polycrystalline diffraction pattern that is belonged to 3C-SiC. High-resolution TEM (HRTEM) image of 3C-SiC nanocrystal in Figure 4c shows the crystal boundaries among three grains with different orientations. After being measured, the interplanar crystal spacing (*d*), as denoted in Figure 4c, is 0.251 nm, that is, in accordance with the *d* value of {111}_3C-SiC_. The inversed FFT (Fast Fourier Transformation) image of HRTEM image in Figure 4c is shown in Figure 4d, which more clearly shows the lattice image of three grains with different orientations.

### 3.2. Microstructure Analysis of ZrO_2_ Thin Film

**Figure 5:**
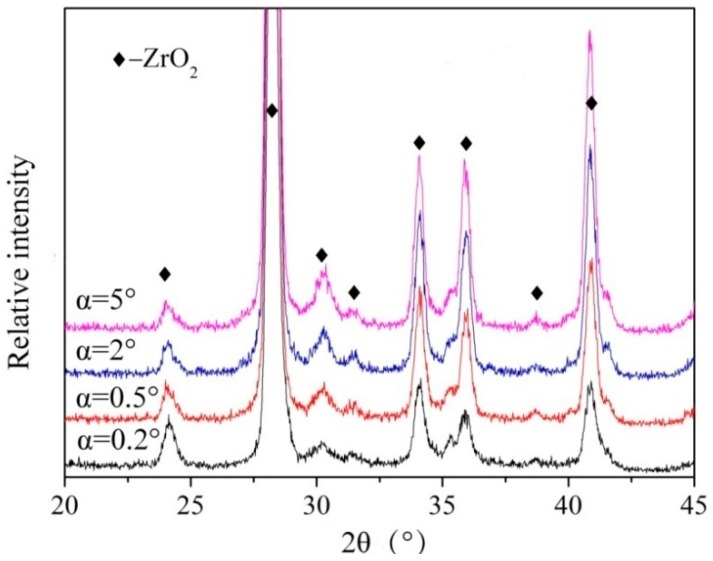
XRD patterns of the annealed ZrO_2_ thin film.

According to the total reflection model [27], the calculated detection depth of X-rays is 270 nm when the incident angle α is 5°, which locates in the zirconium oxide film. The GIXRD characterization results at the incidence angles ranged from 0.2° to 5° of the annealed sample reveal that the main component is monoclinic ZrO_2_ (JCPDS#65-1023 Monolinic ZrO_2_, main peak with the (-111) orientation 2θ = 28.181°). It can be seen that, except for the diffraction strength, the positions of diffraction peaks are almost similar, as shown in Figure 5. It can be concluded that the phase in the thin film is monoclinic ZrO_2_.

**Figure 6:**
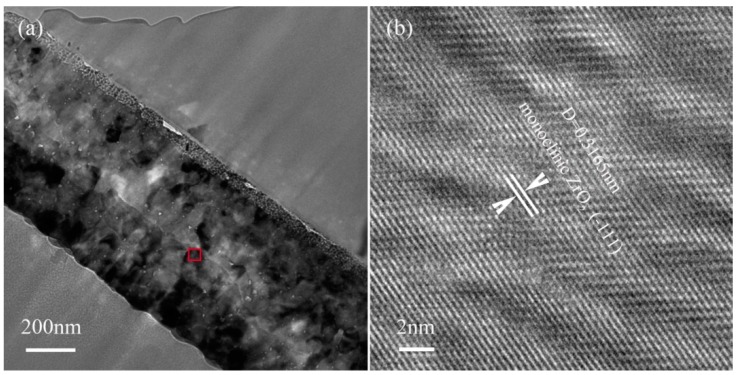
Bright field TEM images of (**a**) annealed ZrO_2_ film and (**b**) HRTEM image of the area marked with a red rectangle in (**a**).

Figure 6a is the cross-sectional bright field TEM image of the prepared ZrO_2_ thin film. It can be seen that the thickness of ZrO_2_ layer is fairly uniform and is about 600 nm. Figure 6b is the HRTEM image of the area marked with red rectangle in Figure 6a. After being measured, the interplanar crystal spacing (*d*), as denoted in Figure 6b, is 0.3165 nm, that is, in accordance with the *d* value of {-111}_ZrO2_. The analysis result of TEM is consistent with the result of XRD. In addition, Moir fringes can also be observed, which is due to the overlapping of two crystal layers with different orientations [39]. In this work, the ZrO_2_ is mainly used as the transition layer between 3C-SiC layer and zirconium alloy fuel cladding.

### 3.3. Microstructure Analysis of 3C-SiC/ZrO_2_ Bilayer Films

**Figure 7:**
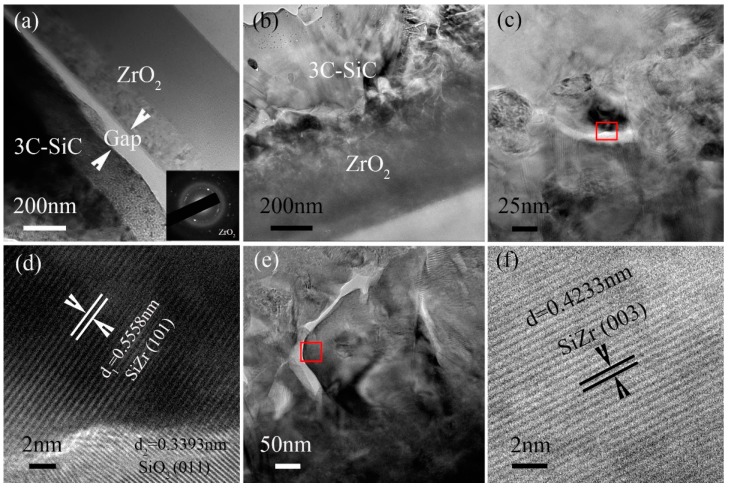
Cross-sectional bright field TEM images of the annealed 3C-SiC/ZrO_2_ thin films, (**a**) Microstructure of T-3C-SiC/ZrO_2_ thin film; (**b**–**f**) Microstructure of O-3C-SiC/ZrO_2_ thin film; (**d**) and (**f**) HRTEM images of the areas indicated by red rectangles in (**c**,**e**), respectively.

From the TEM image shown in Figure 7a, a gap with approximately 100 nm between 3C-SiC layer and ZrO_2_ layer can be observed in the T-3C-SiC/ZrO_2_ thin film. The binding force between 3C-SiC and ZrO_2_ is very weak, which will be separated under a low external load and, further, is difficult in practical application. However, the cross-sectional microstructure of O-3C-SiC/ZrO_2_ thin film, as shown in Figure 7b, indicates a compact combination between 3C-SiC and ZrO_2_, although the interface is no clear. The binding force between 3C-SiC layer and ZrO_2_ layer should be strong. The high magnification images of the interface of the O-3C-SiC/ZrO_2_ thin film are shown in Figure 7c,e. The HRTEM images taken from the red rectangles are shown in Figure 7d,f. The analysis results indicate that the compounds of SiZr and SiO_2_ are synthesized at the interface. In Figure 7d, the lattice images include SiZr with (101) orientation and SiO_2_ with (011) orientation. Also, Figure 7e shows the lattice image of SiZr (003). During the preparation process, Zr layer was first deposited on Si substrate, and then SiC layer was deposited on the surface of Zr layer. After being deposited, amorphous SiC/Zr bilayer films were finally annealed to form nanocrystal structures in a tube furnace.

Because of the argon protection with low-speed flow and the tube furnace with low gas tightness, a small amount of oxygen sneak into the sintering system. As the oxidizability of amorphous Zr is much easier than that of amorphous SiC, these small amount of oxygen will be first reacted with amorphous Zr to form crystal ZrO_2_ during the 1000 °C annealing process. Additionally, a tiny amount of oxygen is reacted with amorphous SiC to form SiO_2_ phase at the interface of 3C-SiC/ZrO_2_ films. Meanwhile, the reaction between Zr and Si is also carried out to form the SiZr phase. Because of the intermediate compounds of SiZr and SiO_2_, the lattice mismatch degree will be obviously decreased between the 3C-SiC layer and ZrO_2_ layer. Furthermore, the binding force between 3C-SiC film and ZrO_2_ film is significantly increased.

### 3.4. Corrosion Results

**Figure 8:**
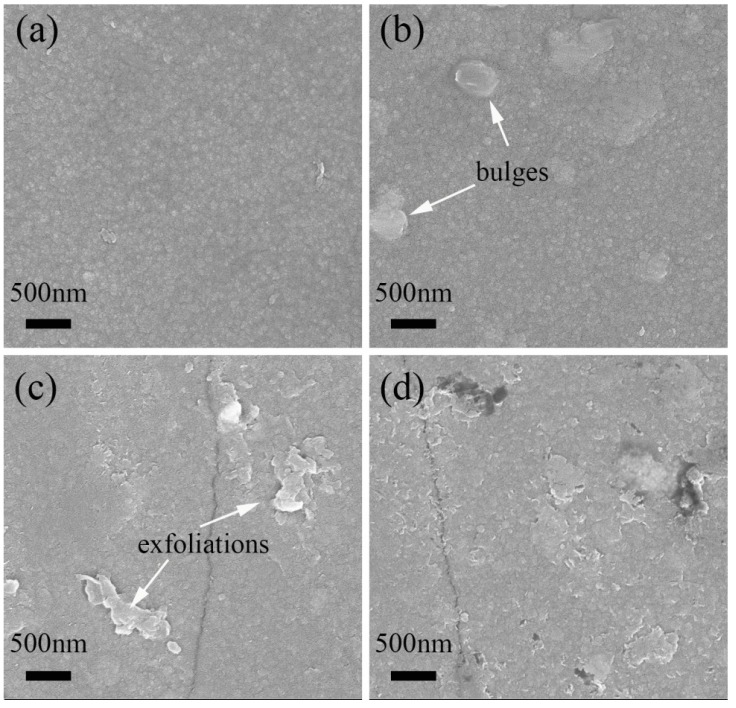
Scanning electron microscopy (SEM) images showing the surface morphology of the prepared 3C-SiC/ZrO_2_ films (**a**) and the 3C-SiC/ZrO_2_ films corroded at 100 °C water bath for 0.5 h (**b**); 10 h (**c**) and 30 h (**d**).

The corrosion experiments were carried out at 100 °C in a water bath and under normal pressure to qualitatively test the surface corrosion resistance and the binding force of the interface of the O-SiC/ZrO_2_ films. The corrosion time was ranged to 30 h. Figure 8 shows the surface morphology of the prepared sample and the corroded samples. The surface of the prepared O-SiC/ZrO_2_ films is relatively flat and has no special characteristics, as shown in Figure 8a. After corrosion for 0.5 h, some bulges appear on the sample surface, as shown in Figure 8b. While after further corrosion for 10 h, as shown in Figure 8c, most exfoliations appear on the sample surface, which is induced by the fracture of the bulges. The cracks also appear on the sample surface, which should be due to the corrosion stress. Although the corrosion time is increased to 30 h, the surface crack dose not grow and the surface exfoliations also have no special change, as shown in Figure 8d. Therefore, the surface corrosion resistance should be great as the surface of the sample shows no significant corrosion evolution phenomenon with the increase of corrosion time. The interface binding force is also great, as there is no mass shedding phenomenon that appeared on the sample surface. In the future, the corrosion behavior under high temperature and high pressure, thermodynamic properties, mechanical properties and radiation resistance will be tested to assess the properties of SiC/ZrO_2_ films.

## 4. Conclusions

Nanocrystal 3C-SiC/ZrO_2_ bilayer films used as a protective coating of zirconium alloy fuel cladding were prepared on a single-crystal Si substrate. The divided investigations of nanocrystal 3C-SiC film and nanocrystal ZrO_2_ film were also carried out. The microstructure of the synthesized nanocrystal films was analyzed by GIXRD and cross-sectional TEM. The following conclusions can be made:

(1) The nanocrystal 3C-SiC thin film with a width of approximately 100 nm was synthesized by PECVD and annealing. The average grain size was less than 30 nm. The optimal preparation parameters were an annealing time of 1.5 h, an annealing temperature of 1000 °C and the ratio of SiH_4_:CH_4_ = 2:10.
(2) An amorphous Zr layer with a width of about 600 nm was first deposited on a single crystal Si substrate by magnetron sputtering and then oxidized to form a ZrO_2_ layer with a crystal structure during the annealing process.
(3) The interface characteristics of the nanocrystal 3C-SiC/ZrO_2_ bilayer films prepared by two different processes were obviously different. There was a gap and a weak binding force between 3C-SiC film and ZrO_2_ film under the preparation technique that first synthesized the crystal ZrO_2_ film and then prepared the crystal 3C-SiC film on the surface of the crystal ZrO_2_ film. However, the strong binding force and the compounds such as SiZr and SiO_2_ were formed at the interface of 3C-SiC/ZrO_2_ bilayer films under the preparation technique that first deposited the amorphous Zr layer, then deposited the amorphous SiC layer, and finally annealed at the argon protection with low-speed flow.
(4) The corrosion resistance of 3C-SiC/ZrO_2_ bilayer films is good after corrosion for 30 h at 100 °C in a water bath and under normal pressure.

## Figures and Tables

**Table 1:** Preparation parameters of the nanocrystal 3C-SiC thin film. Radio frequency (RF).

Code	SiH_4_ (sccm)	CH_4_ (sccm)	Deposition Pressure (Torr)	RF Power (W)	Si Substrate Temperature (°C)	Annealing Temperature (°C)
#1	2	10	0.2	100	400	1000
#2	3	8	0.2	100	400	1000
#3	4	6	0.2	100	400	1000
#4	5	5	0.2	100	400	1000

**Table 2:** Preparation parameters of nanocrystal ZrO_2_ thin film. (DC: Direct Current).

Target	Base Pressure (Torr)	Working Pressure (Torr)	DC Power (W)	Ar (sccm)	Deposited Time (Min)	Annealing Temperature (°C)	Annealing Time (h)
Zr	1.6 × 10^−5^	5 × 10^−3^	150	10	90	1000	1.5

**Table 3:** The parameters of chemical bonds.

Bond Type	Energy (kJ/mol)	Length (Pm)
Si–H	318	148
Si–C	318	185
Si–Si	222	233
C–H	411	109
C–C	346	154
H–H	432	74
